# Challenges Faced by U.S. Veterinary Technicians in the Workplace During COVID-19

**DOI:** 10.3389/fvets.2022.831127

**Published:** 2022-03-07

**Authors:** Zoe C. Rowe, Merritt L. Drewery, Ryan G. Anderson, Crystal M. Russo

**Affiliations:** Department of Agricultural Sciences, Texas State University, San Marcos, TX, United States

**Keywords:** pandemic, burnout, mental health, attrition, personal protective equipment, PTSD, veterinary nurses

## Abstract

During COVID-19, the demand for veterinary technicians increased due to increased animal care appointments booked, decreased worker productivity, pandemic-related staffing shortages, and adapted methods of care delivery. Research has been conducted to assess the effect of the COVID-19 pandemic on educators and human healthcare workers, but there is a lack of literature on veterinary technicians, the animal healthcare equivalent of nurses. The objective of our study was to evaluate how COVID-19 affected veterinary technicians. We distributed an electronic researcher-developed survey-based instrument to veterinary technicians working in the U.S. during COVID-19. We received 1,132 usable responses. Descriptive statistics were analyzed using SPSS 26.0. Our respondents were overwhelmingly female (97%) and mostly employed full-time (87%) in a companion animal practice (61%). A majority of respondents reported COVID-19 had a large effect (45%) or completely dominated the work (12%) at their practice. While 52% of respondents felt their efforts during COVID-19 were appreciated, only 43% agreed or strongly agreed their hours were manageable. Support staff availability was completely or barely adequate for 42% of respondents and personal protective equipment was mostly or completely adequate for 60% of respondents. The greatest professional challenges during COVID-19 were being treated worse by animal owners and difficulty communicating with clients (53 and 16% of respondents, respectively). There have been few efforts to document the professional environment experienced by veterinary technicians during COVID-19. This is critical as pre-pandemic data indicate veterinary technicians are high-risk for professional burnout and COVID-19 placed additional burdens on essential workers.

## Introduction

On December 11th, 2019, the World Health Organization initiated actions to mitigate the spread of the novel coronavirus caused by SARS-CoV-2. By January 21^st^, 2020, the Centers for Disease Control (CDC) confirmed the first case of COVID-19 in the United States and, by mid-March 2020, Americans were given stay-at-home orders to slow the spread of the virus (1). Most professional interactions and operations abruptly moved to remote or online platforms. However, some workers were deemed “essential” and continued working under CDC regulations and guidance. From an animal care perspective, the increased amount of time the general public spent at home increased pet ownership (2), causing a spike in new pet owners during COVID-19.

Before COVID-19, veterinary professions were in demand; the 2019-2029 job outlook for veterinary technologists and technicians was 16%, which was much greater than the average for all occupations, 4% (3). The COVID-19 pandemic likely exacerbated the need for veterinary workers due to the increase in appointments booked, decrease in productivity among veterinary workers, staffing shortages related to the pandemic, and adapted methods of care delivery (4, 5). In veterinary medicine, veterinary technicians, also referred to as “veterinary nurses,” are the equivalent of human healthcare nurses although there is a disparity in pay: the 2020 median pay for veterinary technicians was $36,260 (3) and for licensed practical and vocational nurses was $48,420 (6). Accordingly, they are often tasked with the “dirty work” (7), work long hours, and experience compassion fatigue (8); these factors contribute to a high burnout rate for the veterinary technician profession (9). Although there has been research on the physical and psychological wellbeing of nurses (10), little research has been conducted on veterinary technicians. Further, there has been a plethora of research on the impact of COVID-19 on essential workers in the education (11) and human healthcare (12) fields, but there have been few evaluations on veterinary technicians.

The purpose of our study was to address the deficit in research focused on veterinary technicians. Although there is literature about veterinary technicians and their emotional well-being as it relates to professional burnout (7–9) and there is literature about how COVID-19 has changed the way veterinary practices operate (4, 13, 14), there have been few efforts to document how circumstances surrounding the pandemic affected veterinary technicians. In a U.K. study conducted in June 2020, the mental wellbeing of equine veterinary technicians had been negatively impacted during COVID-19 as compared to previous years, and the extent of this impact was greater than for veterinary surgeons (15). Accordingly, the objective of our study was to characterize the professional environment, faced by veterinary technicians during COVID-19.

## Materials and Methods

This study employed a mixed methods approach to data collection, facilitated through an electronic survey-based questionnaire. The questionnaire was designed to assess how the COVID-19 pandemic affected the professional experience of veterinary technicians. The Texas State University Institutional Review Board approved this research as exempt (#7591) and all participants were provided written informed consent prior to participation. The population of interest was adults (18+ years of age) in the United States who were employed as veterinary technicians during the COVID-19 pandemic. Using a population of 112,900 veterinary technicians (3), a sample size of 1,073 with a 99.9% confidence level and ± 5% confidence interval was calculated.

Our survey was distributed via convenience sampling. Specifically, we posted it on social media platforms (e.g., Facebook, Reddit) to groups targeted toward veterinary workers and sent it to societies and clubs for veterinary workers at the certification, collegiate, and professional levels. The survey was a researcher-developed instrument that contained three sections preceded by three “dummy” questions and ReCAPTCHA technology that ensured respondents were not bots. An example dummy question is: “Do you eat rocks?” where the response “Yes” would send the respondent to the end of the survey without displaying additional questions. Section 1 of the survey consisted of 17 questions including personal and professional demographics. Section 2 contained 14 questions related to the professional experiences of veterinary technicians during COVID-19. Section 3 consisted of four questions related to daily operations of a veterinary practice, how they have changed during the COVID-19 pandemic, and how this change has affected veterinary technicians. The select data presented here are from Sections 1, 2, and 3.

Following recommendations on establishing a face-validated instrument (16), we identified a panel of experts outside of the research team and participant group. The panel included ten individuals with expertise in survey design and/or veterinary professions. The panel assessed the questionnaire for face, content, and construct validity. Based on initial panel recommendations, we revised the questionnaire and resubmitted it for further review until the final version was approved.

To establish reliability, the questionnaire was piloted by veterinary technicians who were not part of the research team, participant group, or expert panel. We sent twelve veterinary technicians a link to the survey. Within seven days, we received ten completed questionnaires, yielding a response rate of 83%. Data from the pilot study were coded and entered using the Statistical Package for the Social Sciences 26.0 software. We calculated a Cronbach's alpha reliability coefficient (α = 0.846) which, based on interpretations in similar research (17), was “good.”

Our questionnaire was available to participants from February 22^nd^, 2021 to March 21^st^, 2021. Ultimately, 1,132 eligible respondents completed at least 88% of the survey. With a response rate exceeding that necessary for our sample, no additional procedures were used to account for non-response error, following previous recommendations (18).

Using SPSS 26.0, data were analyzed using descriptive statistics and measures of central tendency to report how the COVID-19 pandemic affected veterinary technician experiences during the pandemic. We also reported how the pandemic affected the professional environment of the practices our respondents were employed at. Additionally, descriptive statistics were calculated for the demographic characteristics of our respondents and their places of employment. Finally, qualitative data were analyzed inductively by two authors to determine themes. The two authors independently familiarized themselves with the data; searched for themes; merged redundant or similar themes to develop meaningful, comprehensive themes; and refined theme names. Both authors then met with a third author to compare results and determine final themes; the third author was a tiebreaker, as needed (19).

## Results

We received 1,569 responses to our survey. Of those responses, 1,426 respondents were not bots, were at least 18 years of age, and had worked as a veterinary technician in the United States during the COVID-19 pandemic and were, thus, eligible. Of those eligible respondents, 1,132 completed at least 88% of the survey and were included in our dataset.

An overwhelming majority of our respondents (97.2%) were female and were 18–34 (56.3%) or 35–40 years of age (34%) (Supplementary Table S1). Most respondents, 76.4%, were not immune compromised nor living with someone who was immune compromised during the COVID-19 pandemic. A slight majority were graduates of an accredited American Veterinary Medical Association (AVMA) program (59%) and had worked as a veterinary technician for <1 year (4%), 1–6 years (41%), 7–15 years (30%), or 16+ years (25%). Most were currently employed as a veterinary technician (97%) and, of those who were not (3%), 50% left the profession due to COVID-19. Most respondents were employed full-time (87%) and held an official title of either Veterinary Technician with a license (58%) or Veterinary Technician without a license (35%).

We also asked respondents about characteristics of the veterinary practice or clinic by which they were employed (Supplementary Table S2). By design, all respondents worked in the United States. Specifically, they were mostly from the West South Central (18%), South Atlantic and Puerto Rico (16%), or East North Central region (14%); those regions with the least respondents were New England (7%) and the East South Central region (5%) (U.S. Census Bureau, 2021). Most respondents worked in a companion animal (61%), emergency (11%), mixed animal (9%), or specialty practice (8%). A slight majority worked in a suburban area (54%) although a fair amount worked in an urban or metropolitan area (32%). Finally, respondents reported working at a practice with 4+ (40%), 3 (21%), 2 (24%), or 0 or 1 (15%) Doctors of Veterinary Medicine (DVMs).

To gauge the extent to which COVID-19 impacted veterinary technicians, we asked about the effect of the pandemic on their practice or clinic, coworkers, and selves (Table 1). Very few (2%) respondents reported that COVID-19 did not affect their practice or clinic. Rather, 30% reported a moderate effect, 45% a large effect, and 12% reported that the pandemic completely dominated their practice or clinic's work. We received a similar response from our respondents when asking about the extent of the effect of COVID-19 on their coworkers: 2% reported no effect, 10% a small effect, 31% a moderate effect, 46% a large effect, and 11% reported the pandemic completely dominated their coworkers' work. When responding about the effect of COVID-19 on themselves, slightly more (5%) respondents reported COVID-19 did not affect them and slightly less (8%) reported COVID-19 completely dominated their work. There was also a shift away from “large effect” (38% of respondents) to “moderate” (36%) or “small” (13%) effect.

**Table 1:** Veterinary technicians' perceptions of the extent of the effect of COVID-19 on work for their practice, coworkers, and selves.

	**No effect**	**Small effect**	**Moderate effect**	**Large effect**	**Completely dominated work**
Practice	2%	11%	30%	45%	12%
Coworkers	2%	10%	31%	46%	11%
Self	5%	13%	36%	38%	8%

We strove to understand the work atmosphere that veterinary technicians experienced during COVID-19. Thus, we asked about the appreciation they received, their work hours, work decisions and priorities, and feelings of support from coworkers or management (Table 2). While 52% of respondents either strongly agreed or agreed that their efforts were appreciated at work during the pandemic, 28% disagreed or strongly disagreed. Further, 38% of veterinary technicians did not feel their hours during COVID-19 were manageable and 26% felt decisions regarding work assignments were not fair. Most respondents (73%) strongly agreed or agreed that they worked within their area of competency during COVID-19. The priorities of the veterinary practices our respondents were employed at were consistent, for the most part, with their own values (41% of respondents agreed or strongly agreed). Overall, 59% of our respondents felt supported by their coworkers during the pandemic with only 16% disagreeing and 6% strongly disagreeing. They also felt confident their decisions were supported by management or superiors with only 23% disagreeing or strongly disagreeing.

**Table 2:** Veterinary technicians' perceptions of their work atmosphere during COVID-19.

	**Strongly agree**	**Agree**	**Unsure**	**Disagree**	**Strongly disagree**
Efforts were appreciated	18%	34%	20%	18%	10%
Hours were manageable	9%	34%	19%	22%	16%
Worked within areas of competency	24%	49%	11%	11%	5%
Decisions for work assignments were fair	10%	42%	22%	19%	7%
Practice's priorities aligned with own values	11%	30%	25%	23%	11%
Felt social support from coworkers	16%	43%	19%	16%	6%
Felt confident that decisions were supported	15%	39%	23%	17%	6%

As COVID-19 demanded specific and additional resources for work, we asked our respondents about the adequacy of their personal protective equipment (PPE), support staff, and information received from management (Table 3). Only 3% of respondents felt their PPE was completely inadequate during COVID-19 with 10% reporting it was barely adequate, 27% reporting it was somewhat adequate, 40% reporting it was mostly adequate, and 20% reporting it was completely adequate. The availability of support staff during COVID-19, however, was completely inadequate or barely adequate for 12 and 30% of our respondents, respectively. Only 8% of respondents reported support staff availability was completely adequate. Competency of support staff during the pandemic was viewed in a more favorable light with 46% of respondents reporting support staff competency was mostly or completely adequate and only 21% reporting it was barely adequate or completely inadequate. The information received from management during COVID-19 was, for the most part, either barely adequate (20% of respondents), somewhat adequate (27% of respondents), or mostly adequate (28% of respondents).

**Table 3:** Veterinary technicians' perceptions of the adequacy of resources they were provided during COVID-19.

	**Completely inadequate**	**Barely adequate**	**Somewhat adequate**	**Mostly adequate**	**Completely adequate**
PPE	3%	10%	27%	40%	20%
Support staff availability	12%	30%	28%	22%	8%
Support staff competence	5%	16%	33%	33%	13%
Information from management	9%	20%	27%	28%	16%

We asked respondents more specifically about how COVID-19 impacted them with an emphasis on routine procedures or situations that veterinary technicians may experience in their daily work (Table 4). Notably, 43% of respondents strongly agreed and 34% agreed that they were treated worse by animal owners during COVID-19. We received a similar response when asking about communication with animal owners; 30% strongly agreed and 45% agreed communication was more difficult during COVID-19. Interestingly, however, 67% of veterinary technicians felt work was easier due to the animal owner not being in the room during animal care, an observation likely attributed to curbside policies implemented by veterinary practices to abide social distancing guidelines during the pandemic. Respondents were split on whether or not animals were more difficult to handle during COVID-19; 34% strongly agreed or agreed, 27% neither agreed nor disagreed, and 39% disagreed or strongly disagreed. However, 45% of respondents felt the PPE required due to COVID-19 seemed to frighten the animals. Fifty-one percent of respondents reported it was more difficult to balance animal care and what the owner could afford during the pandemic. A majority (65%) experienced difficulty ensuring personal safety during euthanasia; this is likely attributed to allowing the animal owner to remain in the room during euthanasia. There was also concern over being sent home sick when money is needed; 50% of our respondents agreed or strongly agreed that this was a concern whereas 43% disagreed or strongly disagreed. Ultimately, when asked if they had considered changing careers since COVID-19, 21% of respondents strongly agreed, 24% agreed, 12% neither agreed nor disagreed, 24% disagreed, and 20% strongly disagreed.

**Table 4:** Veterinary technicians' perceptions of their routine, work-specific experiences during COVID-19.

	**Strongly agree**	**Agree**	**Neither agree nor disagree**	**Disagree**	**Strongly disagree**
Treated worse by animal owners	43%	34%	16%	5%	2%
Animals were more difficult to deal with or handle	12%	22%	27%	29%	10%
More difficulty balancing animal care and affordability	12%	39%	32%	15%	2%
Concerned over being sent home sick when money is needed	21%	29%	17%	22%	11%
Considered changing careers since COVID-19	21%	24%	12%	24%	20%
Work was easier due to owners not being in the room during animal care	32%	35%	20%	10%	3%
Work was more difficult due to PPE for COVID-19	15%	40%	19%	20%	6%
PPE due to COVID-19 seemed to frighten animals	11%	34%	27%	21%	7%
More difficult communicating with animal owners	30%	45%	14%	10%	1%
Difficulty ensuring personal safety during euthanasia	28%	37%	16%	15%	4%

When asked about the greatest challenge at their practices or clinic during COVID-19, 53% of our respondents reported that it was being treated worse by pet owners. Others reported difficulties communicating with pet owners (16%); “other,” which allowed them to respond to an open-ended prompt (15%); balancing pet care and cost (5%); maintaining personal safety during euthanasia (4%); the PPE making work duties more difficult (4%); pets being more difficult to deal with (2%); and the PPE frightening pets (1%). For respondents who selected “Other” (15%, *n* = 170), common themes identified were: (1) owners would not abide by curbside policies (55%), (2) practices were understaffed and veterinary technicians were overworked (32%), and (3) ineffective or unempathetic management/leadership (13%).

## Discussion

Our study characterized the professional environment faced by veterinary technicians during the COVID-19 pandemic. Our findings demonstrate that veterinary technicians experienced additional work stressors and challenges during COVID-19. Specifically, communication with and treatment by animal owners during COVID-19 was a challenge, in addition to the number of hours required to work and availability of support staff.

Before COVID-19, veterinary technicians experienced high rates of burnout due to compassion fatigue, excessive workload, lacking a sense of autonomy, limited resources, and doing the “dirty work” (7–9). The healthcare industry tends to attract compassionate, empathetic people. However, these empathetic people are also those who are most affected by witnessing others, including animals, suffer from disease and death (7). Further, the lack of upward mobility and low compensation may cause veterinary technicians to eventually pursue other career paths (8), resulting in high turnover for those in veterinary careers.

Circumstances surrounding COVID-19 proved difficult for many, whether they were “essential workers” or not. Unemployment and suicide for the general population skyrocketed (20). The pandemic also demanded constant flexibility; essential workers had to adapt to ever-changing policies and procedures as health professionals learned more about the virus and changed guidance. During the pandemic, healthcare workers exhibited more psychopathological symptoms (e.g., severe depression, anxiety) as compared to other professions (21). Further, essential workers were also burdened with the risk of being exposed to COVID-19. Veterinary clinics and practices shifted to curbside services, encouraged clients to receive consultations via telehealth conferences, and/or split teams into shifts to lower the risk of all employees becoming infected with COVID-19 at once (4, 13). Parallel to these operational shifts, there was an increase in owners euthanizing their animals due to financial uncertainty (13), suggesting that veterinary technicians had to more often participate in work tasks that are emotionally taxing as a result of COVID-19.

Although it is unknown if veterinary practices or clinics were actively working to reduce the above cited burnout factors before COVID-19, it is apparent from the present and previous data (4, 22) that conditions surrounding the pandemic created additional stress and challenges for veterinary technicians. Specifically, our data indicate that, during COVID-19, veterinary technicians were challenged with inadequate support staff availability, unmanageable work hours, communication with and treatment by animal owners, and COVID-19-specific PPE making work duties more difficult. These challenges are echoed in similar work conducted during the pandemic (14) which demonstrated that veterinary workers – mostly veterinarians – experienced an increase in the frequency of ethically challenging situations and unique stressors during COVID-19.

During times of acute clinical need, nurses and other health professionals often continue working, deferring grief until after the need has passed; this ultimately heightens their risk of burnout and post-traumatic stress disorder (PTSD) (23). The risk of burnout, PTSD, and compassion fatigue are further compounded by engaging nurses in critical care environments when they are not equipped with critical care training or experience (24). Arguably, the veterinary technicians in our study were not equipped to balance the new professional duties COVID-19 demanded, especially as they relate to customer relationships and communication, while also protecting themselves from COVID-19 and managing their own mental health. Our data, in addition to that of others (14, 15), indicate the pandemic placed new and additional pressures on veterinary technicians, workers who already experience high levels of emotional distress and burnout (9). This underlines the need for additional physical and psychological support for veterinary technicians. In a study focused on the resilience of nurses during the pandemic, those who perceived higher organizational support were more likely to report lower anxiety related to COVID-19 (25). These researchers recommended that nurse managers ensure nurses are given access to psychological treatment; should prioritize self-care by offering flexible or shorter work hours, adequate breaks, and balanced time scheduling; and provide nurses with complete and quality PPE (25). Ultimately, we recommend managers of veterinary technicians adopt a similar approach during the COVID-19 pandemic and similar events that disrupt society (e.g., terroristic attacks, major climatic events, wildfires), with a strong focus on mental and physical health. Our respondents did, overall, feel confident their decisions were supported and that their efforts at work were appreciated, indicating that support is being received from their practice or clinic. Further, they reported receiving social support from their coworkers, a promising finding.

In Australia, nurses working during the pandemic reported a need to perform duties outside of their normal routines, including cleaning and answering calls (12). Similarly, in other research focused on nurses in the U.K. (26), 57% experienced a change in their role during the pandemic. Of Australian nurses who continued to perform routine work duties, few of them had prior experience in disaster relief or contact training and felt unprepared (12). Our data contrast those for nurses – 73% of our respondents either agreed or fully agreed that they worked within their area of expertise during COVID-19. While an improvement over their human healthcare counterparts, this still indicates the pandemic required our veterinary technician respondents to work outside of their area(s) of expertise, which could have implications for both human and animal safety.

A common theme in our data was that veterinary technicians were treated worse by animal owners during COVID-19. Veterinary technicians are often the liaison between a veterinarian and the animal owner and are, thus, placed in a position to receive blame but not praise. Animal owners might have treated veterinary technicians worse during the pandemic because they, themselves, were experiencing psychological stress and anxiety or were afraid of the costs of animal care due to the higher rate of unemployment during COVID-19 (27). During the pandemic, there was concern about the availability of veterinarians; these perceptions of scarcity could have caused animal owners to lash out and become more irritated during their appointment or communication with veterinary technicians (28). Further, since many veterinary practices or clinics used curbside services and did not allow non-employees in the building during COVID-19 (4), owners were not likely allowed to be in the room with their animal during care and this may have caused distress or heightened anxiety, especially if the outcome was fatal. Our respondents reported this separation as a favorable aspect of the pandemic; the majority agreed that not having the animal owner in the room made their work easier, although they acknowledged that this was at the sacrifice of ease of communication with the owner. Veterinary staff harassment by and difficulty communicating with animal owners during COVID-19 are known challenges (29, 30) previously attributed to disgruntlement over adherence to imposed safety protocols (29). It was previously recommended that clinic owners and mangers be mindful of these challenges and follow the lead of human healthcare facilities to protect staff (29).

The pandemic impacted the demand for and access to PPE as there was a sudden and significant need for the general population to access PPE, such as masks and gloves, typically reserved for the healthcare industry. Australian nurses expressed concern about PPE during COVID-19, as only 27% reported “always” having enough gowns and 23% reported “always” having enough P2/N95 masks available (12). Access to PPE in the U.K. seemed to be slightly better than in Australia as only 28% of U.K. nurses were concerned about not having enough PPE during the pandemic (26). It should be stated, however, that this percent (28%) is still large and underlines a massive disparity between the need for and availability of PPE during COVID-19 to protect the physical health of essential workers while they performed work duties. Sixty percent of our respondents felt their PPE was either mostly or completely adequate. This is comparable to another study on veterinary workers during the pandemic (14) in which it was reported that 46% of respondents had to make challenging decisions about PPE distribution at least weekly or several times a month. Further, in April 2020, 41% of U.S. veterinary clinics in asked staff to limit use of PPE as a precautionary measure and, as of July 2020, availability of PPE continued to be a challenge (4). As with our data about working outside of one's area of expertise, our data indicates veterinary technicians experienced an improvement over their human healthcare counterparts, but still faced a deficit in PPE for their job duties which could have negatively impacted their health and wellbeing.

This study is limited as our data were obtained from a convenience sample using an electronic survey-based questionnaire and individual interviews were not attempted. However, this represents the first targeted attempt to characterize the professional environment experienced by veterinary technicians during the pandemic and adds to a growing repository of literature documenting the effect of COVID-19 on essential workers. During and beyond pandemics or similar periods of societal disruption, it is vital that all essential workers are provided the resources they need to fulfill their professional obligations without negatively affecting their physical or mental health. For veterinary medicine, it is imperative that managers of veterinary technicians are aware of the burnout symptoms plaguing veterinary technicians and take measures to prevent them by providing adequate staffing, reasonable work hours, and mental health support. Healthcare workers in the human and animal fields should be trained to identify signs of emotional distress in themselves and their coworkers and peer support groups should be established (29). Finally, animal owners should recognize that COVID-19 protocols implemented by the clinic were developed to ensure health and safety for themselves, their animals, and veterinary workers and should abide by these protocols. Specific guidance for animal owners during COVID-19 has been developed (29); this guidance emphasizes using telehealth and phone calls to receive advice about animal care, as possible, and maintaining social distance if one must take their animal to the clinic.

## Data Availability Statement

The original contributions presented in the study are included in the article/Supplementary Material, further inquiries can be directed to the corresponding author.

## Ethics Statement

The studies involving human participants were reviewed and approved by Texas State University Institutional Review Board. Written informed consent for participation was not required for this study in accordance with the national legislation and the institutional requirements.

## Author Contributions

ZR designed and disseminated the survey instrument, analyzed data, and contributed to writing the manuscript. MD designed the experiment, analyzed and interpreted data, and contributed to writing the manuscript. RA designed the experiment and analyzed data. CR disseminated the survey instrument and contributed to data interpretation. All authors read and approved of the final manuscript. All authors contributed to the article and approved the submitted version.

## Funding

The authors thank the Texas State University College of Applied Arts Learning Communities Grant and USDA National Institute of Food and Agriculture (grant 2020-01979) for funding.

## Conflict of Interest

The authors declare that the research was conducted in the absence of any commercial or financial relationships that could be construed as a potential conflict of interest.

## Publisher's Note

All claims expressed in this article are solely those of the authors and do not necessarily represent those of their affiliated organizations, or those of the publisher, the editors and the reviewers. Any product that may be evaluated in this article, or claim that may be made by its manufacturer, is not guaranteed or endorsed by the publisher.
