# Supplementary material for: Challenges Faced by U.S. Veterinary Technicians in the Workplace During COVID-19
Source: Front Vet Sci. 2022 Mar 7;9:831127. doi: 10.3389/fvets.2022.831127 (PMC8936168; doi:10.3389/fvets.2022.831127)
Supplement: Supplementary file 1 [file Table_1.docx]

| Table S1. Demographics of survey respondents | | |
| --- | --- | --- |
|  | Frequency | Percent |
| Gender |  |  |
| Female | 1,096 | 97% |
| Male | 29 | 3% |
| Graduated from an AVMA program |  |  |
| Yes | 674 | 59% |
| No | 458 | 41% |
| Age range |  |  |
| 18-34 | 637 | 56% |
| 35-50 | 388 | 34% |
| 50+ | 107 | 10% |
| Immune compromised? |  |  |
| Yes | 267 | 24% |
| No | 864 | 76% |
| Years working as a veterinary technician |  |  |
| <1 | 45 | 4% |
| 1-6 | 459 | 41% |
| 7-15 | 339 | 30% |
| 16+ | 288 | 25% |
| Currently employed as a veterinary technician? |  |  |
| Yes | 1094 | 97% |
| No | 30 | 3% |
| If no to above, was it related to COVID-19? |  |  |
| Yes | 15 | 50% |
| No | 15 | 50% |
| Employment status |  |  |
| Full Time | 985 | 87% |
| Part Time | 145 | 13% |
| Official title |  |  |
| Veterinary Assistant | 77 | 7% |
| Veterinary Technician (license) | 660 | 58% |
| Veterinary Technician (no license) | 392 | 35% |

| Table S2. Demographics of survey respondents’ places of employment | | |
| --- | --- | --- |
|  | Frequency | Percent |
| Practice location in the U.S. (region)* |  |  |
| Pacific | 133 | 12% |
| Mountain | 83 | 8% |
| West North Central | 92 | 8% |
| East North Central | 163 | 14% |
| West South Central | 203 | 18% |
| East South Central | 53 | 5% |
| South Atlantic and Puerto Rico | 180 | 16% |
| Middle Atlantic | 139 | 12% |
| New England | 72 | 7% |
| Field of veterinary medicine |  |  |
| Animal Shelter | 34 | 3% |
| Companion Animal Practice | 688 | 61% |
| Emergency Practice | 128 | 11% |
| Equine Practice | 18 | 2% |
| Mixed Animal Practice | 102 | 9% |
| Non-Profit | 19 | 2% |
| Specialty Practice | 92 | 8% |
| University | 34 | 3% |
| Other | 15 | 1% |
| Description of practice location |  |  |
| Rural or farm | 164 | 14% |
| Suburban | 603 | 54% |
| Urban or metropolitan | 1122 | 32% |
| Number of DVMs at practice |  |  |
| 0 or 1 | 167 | 15% |
| 2 | 271 | 24% |
| 3 | 239 | 21% |
| 4+ | 453 | 40% |
| *As defined by [32] | | |

32. U.S. Census Bureau. Census Bureau regions and divisions within state FIPS codes (2021). Available online at: https://www2.census.gov/geo/pdfs/mapsdata/maps/reference/us_regdiv.pdf (accessed April 27, 2021).
